# With Great Power Comes Great Responsibility: the Importance of Rejection, Power, and Editors in the Practice of Scientific Publishing

**DOI:** 10.1371/journal.pone.0085382

**Published:** 2013-12-30

**Authors:** Christopher J. Lortie, Stefano Allesina, Lonnie Aarssen, Olyana Grod, Amber E. Budden

**Affiliations:** 1 Department of Biology, York University, Toronto, Ontario, Canada; 2 Department of Ecology & Evolution, The University of Chicago, Chicago, Illinois, United States of America; 3 Department of Biology, Queen’s University, Kingston, Ontario, Canada; 4 National Center for Ecological Analysis and Synthesis, The University of California Santa Barbara, Santa Barbara, California, United States of America; Université de Montréal, Canada

## Abstract

Peer review is an important element of scientific communication but deserves quantitative examination. We used data from the handling service manuscript Central for ten mid-tier ecology and evolution journals to test whether number of external reviews completed improved citation rates for all accepted manuscripts. Contrary to a previous study examining this issue using resubmission data as a proxy for reviews, we show that citation rates of manuscripts do not correlate with the number of individuals that provided reviews. Importantly, externally-reviewed papers do not outperform editor-only reviewed published papers in terms of visibility within a 5-year citation window. These findings suggest that in many instances editors can be all that is needed to review papers (or at least conduct the critical first review to assess general suitability) if the purpose of peer review is to primarily filter and that journals can consider reducing the number of referees associated with reviewing ecology and evolution papers.

## Introduction

Peer review by editors and referees can improve science and publications. Time and effort, in addition to significant delays in the dissemination of important findings, are real costs to academic scientists that are potentially paid unequally [1] and may not benefit all authors universally [2]. In a recent study exploring this topic, the authors imply that rejection and subsequent revision improves manuscript performance because resubmitted manuscripts were ultimately more cited [3]. An extended interpretation to this finding is that we should ‘reject more’ [4] as this impacts the supply-demand ratio for a journal or at least the perception thereof. Ethical considerations aside, an alternative interpretation is that “increasing revisions, not rejections” can increase the final citation rate and presumed quality [5]. However, perhaps the least attractive but most parsimonious alternative is that on average peer review does not impact the merit of manuscripts at all. In an online comment associated with Ball (2012), it was proposed that no benefit is really needed in reviews because the decisions by authors associated with rejection and attrition alone could generate the pattern detected, i.e. more work on a manuscript improves it and selecting the appropriate journal may occur only after authors try for higher tier options. In our experience, having greater time to reflect on a manuscript whilst waiting for reviews also generates improvements because revisiting the work weeks, or even months later, often leads us to read it more objectively – we speculate - even without external input. In many respects, the science described is fundamentally unchanged, simply the packaging improved with revisions. Furthermore, an agent-based model recently demonstrated that ‘rational’ referees can even deteriorate manuscript quality by differentially supporting networks of collaborators when reviewing [6]. Hence, assuming that peer review has only positive effects on science without examining other alternatives fully with the large publication and citation datasets now accumulating online is naïve. Importantly, given the commendable sample size of the work by Calcagno et al., 80,748, patterns will emerge but may not imply causation. With great statistical power comes pattern. The purpose of this study is to a provide an independent, direct examination of whether there is correlative evidence that additional reviews improve ultimate citation rates and to contrast editor-only reviewed instances with those manuscripts sent out for external review to cursorily explore the relative importance of referees.

The realized power and impact of editors in shaping published science is likely profound. Most work on these topics deals with only the tip of the iceberg, i.e. the final publications, whilst the vast bulk of scientific work in manuscript form is likely still in circulation or some proportion permanently unpublished. Surveys of authors are certainly an excellent solution to this problem [3] but likely not without limitations such as selective reporting/recall. As a test of the importance of peer review and expert-editorial opinion, we found another solution. We secured permission from Manuscript Central (MC, a dominant submission and tracking online system adopted by many journals) to access their database associated with the handling of manuscripts for 10 mid-tier ecology and evolution journals that granted permission but wished to remain anonymous. Using the review history of all manuscripts, we explored the citation success of the accepted instances to test whether additional input in the form of number of reviews correlated with improved citation performance. Given that editors also handle immense volumes of manuscripts and are often senior scientists, we considered the performance of papers reviewed only by editors to explore whether editor-only review is a viable peer-review model.

## Methods

We selected manuscripts submitted and reviewed in 2007 that were ultimately accepted to ensure both an adequate citation window and to track performance as publications. The impact factor of the journals that provided access to the MC database ranged from 1.7 to 7 (2012 impact factor scores) with a mean value of 3.2 +/- 0.6 (1 standard error). A total of 1154 standard research manuscripts were accepted by these journals in this year. Reviews, commentaries, notes, replies, and editorials were excluded. Using Scopus, we located all final publications and associated citations. Given that all manuscripts were published in the same year, total citations were used. The MC database provided both the number of reviews requested and completed but not handling time in a meaningful way. Requested reviews ranged from zero (editor accepted the manuscript without sending out for external review) to nine requests. However, accepted manuscripts in this dataset never had more than 3 external reviews completed. A generalized linear model was used to test for differences between journals and successful number of reviews completed on the total number of citations accrued. A Spearman’s nonparametric correlation analysis was presented *post hoc* to show the relationship between number of reviews and citations [7], and each journal was also examined independently to ensure aggregation did not mask potential relationships [8].

## Results

**Figure 1:**
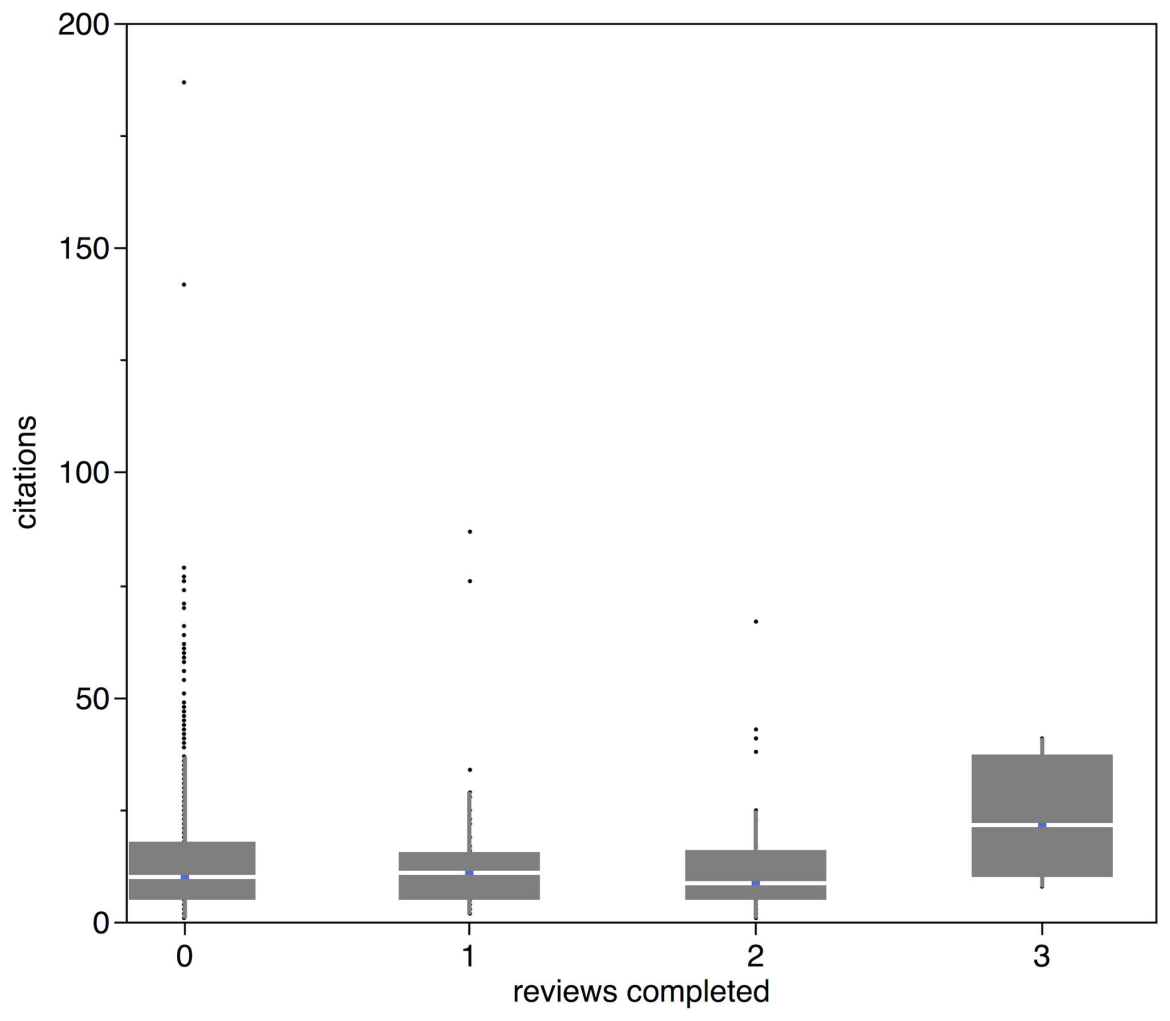
The number of reviews completed for manuscripts from 10 mid-tier ecology and evolutionary biology journals handled in 2007 by total citations (all published in same year). The value of 0 reviews are those manuscripts listed in manuscript central that were accepted and reviewed by only the editor. Comparative box plots are provided with upper and lower quartiles denoted by whiskers and median via a solid line within each box.

The median number of external reviews completed by referees was 2 for this set of manuscripts. There was no relationship between number of reviews completed (both by editors and external referees) and final citations (Figure 1, Generalized Linear Model with journal treated as a random factor, Chi-square _# reviews_ = 0.32, p = 0.57 and Spearman’s non-parametric aggregated *post hoc* correlation analysis of reviews and citations, rho = 0.007, p = 0.8). There was also no significant relationship between citations and the total number of external referees invited (Generalized Linear Model, Chi-square = 0.27, p = 0.6). There were however significant differences between journals in the citations their respective papers accrued (Generalized Linear Model, Chi-square _journal_ = 216.1, p = 0.0001), but independent correlation analyses between reviews and citations were not significant within each set of manuscripts for each journal (Spearman’s non-parametric correlation analyses, all p > 0.4).

## Discussion

Publication is a critical step in the modern scientific process. Papers and reviews are not only a means to disseminate findings but also solicit feedback [9], promote discussion [10], and provide the substrate for new ideas [11] and synthesis [12,13]. In some respects, scientific findings do not exist until they are written and shared in some form [14]. Consequently, peer review has the capacity to improve science, but like every other aspect of the process, including even how we chose to publish findings, it should be open to experimentation [14-16]. Given that there is resistance to change [17] and that there are likely significant shortages in available external referees in ecology and evolution in particular [18], careful examination of the merits of peer review are required. The criteria that we apply to assigning merit is challenging [19,20] and one set of solutions is to delineate *a priori* the standards for the journal and editorial boards such as extent of novelty but this can be flawed [21] or technical correctness and strength of experimental design, i.e. PLOSONE, that has been highly effective. Nonetheless, both options are predicated upon external review. There is another solution – reduced reliance on external reviews. The primary purpose here was to explore whether there was evidence that additional input on manuscripts improved at least the visibility of the final publications in terms of citations. There was no evidence in the dataset examined that additional exposure of a manuscript to reviews related to its ultimate citation rate. Included in these analyses were reviews by editors only and these also did not differ in the citations accrued relative to those sent out for review. Consequently, a parsimonious interpretation of these findings is that peer review either within or across journals does not necessarily improve the likelihood that others use it in subsequent publications. This does not imply that peer review does not improve the manuscript in some form, but it does suggest that external reviews do not significantly alter the work in noticeable ways that increase visibility to others.

The median and maximum number of reviews for the ten mid-tier ecology and evolution journals examined was also informative. In our experience, these findings were representative of most ecological and evolutionary biology journals with two external reviews successfully provided for a given manuscript. At PLOSONE, accepted manuscripts have been reviewed on average by 1.9 external referees (n = 1837 manuscript in 2010, http://www.plosone.org/static/information). Hence, there is every indication that the pool of manuscripts analyzed herein are representative of the natural sciences in terms of willingness by referees to consider them and for editors to decide when review input was sufficient prior to acceptance. Most, if not all, editors in ecology are also authors and appreciate the need for timely reviews themselves [22]. Ecological editors also value speed of review in external referees and quality of previous reviews [22]. However, depending on the purpose of the external reviews solicited (i.e., improvement versus confirmation that the research is sound or appropriate), we speculate that editors may be able to accelerate the dissemination of scientific findings even more rapidly for this discipline by carefully considering when and why reviews are needed. Reuse of reviews between journals has also been proposed as a solution along these lines [15]. Alternatively, editors and journals could consider soliciting different forms of peer review from various external referees such as commentary and evaluation of methods, visualization, or specific suggestions on improvement of the communication. We speculate that this may also have the additional benefit of referee specialization, much like researchers, with individuals developing critical skills in one aspect of review such as statistics, graphics, methodology, etc. Micro-annotation of specific elements of a paper online and transparent commenting on pre-prints such as the efforts by PeerJ (https://peerj.com) are also viable alternatives to traditional peer review. The maximum number of reviews successfully secured for these 10 journals was 3 also suggesting that in spite of sometimes-large numbers of requests, there is a workload carrying capacity per manuscript that most active scientists can sustain. The referee shortage [18] is unlikely to improve whilst the pressure to publish is also increasing. Rejection certainly does not improve science or our breadth of knowledge but reviews can. Similar to many similar recent efforts with large datasets on this topic, correlation is not causation and peer review has many other likely benefits outside visibility of final paper but clear floor and ceiling effects are evident in terms of recognition by peers over the 5-years time period tested here. Editor-only review generates a pool of manuscripts that perform equally well in terms of citations regardless of external reviews. Perhaps, far fewer referees are needed at least for this express purpose. We speculate that this finding is not a call to disregard input from others prior to publication but that reviews do not improve potential visibility once in print.

## Conclusions

We recognize this is a more limited albeit focused examination of the importance of extent of review relative to a previous related study by Calcagno et al., and a slightly countervailing approach in that we examined only those that were accepted and successfully navigated reviewing. All else being equal, we expected that increased input within this pool of manuscripts should have changed some elements attractive to other authors in deciding which papers to cite. We speculate that editors in ecology are very accurately identifying the papers that will advance the field and external reviews may not be changing those perceived impacts. There is at least one very reasonable explanation for this hypothesis. Peer review does improve some aspects of quality but not those that matter to ecologists, either editors, or readers in selecting studies to cite. Ecologists are more interested in quantities such as effect size estimates than quality [23]. Writing, visualizations, style, and clarity are of course all very important. Nonetheless, most scientists are interested in the findings themselves, i.e. the results. The interpretation and context are important but when reading empirical studies we inspect the presentation and analyses of the data as best we can. These attributes may rarely be impacted by traditional peer review. The PLOSONE model is a perfect example of an approach to review for criteria associated more directly with the findings and execution. The time is ripe now for formal experimentation of peer review by ecological editorial boards including reducing the number of reviews, whether they are blinded even to the board, and of course furthering any initiative associated with publishing data directly. After all, with great power comes responsibility, and down weighting the importance of citations, increasing the prominence of the findings themselves, and using peer review to improve multiple aspects of a manuscript will further improved communication and dialogue in the sciences.
